# Preventive care practices to address health behaviours among people living with mental health conditions: A survey of Community Managed Organisations

**DOI:** 10.1016/j.pmedr.2021.101495

**Published:** 2021-07-15

**Authors:** Lauren Gibson, Tara Clinton-McHarg, Magdalena Wilczynska, Joanna Latter, Kate Bartlem, Corinne Henderson, John Wiggers, Andrew Wilson, Andrew Searles, Jenny Bowman

**Affiliations:** a School of Psychological Sciences, College of Engineering, Science & Environment, University of Newcastle, Callaghan, NSW, Australia; b The Australian Prevention Partnership Centre (TAPPC), Sax Institute, Ultimo, NSW, Australia; c Hunter New England Population Health, Hunter New England Local Health District, Wallsend, NSW, Australia; d School of Medicine and Public Health, Faculty of Health and Medicine, The University of Newcastle, Callaghan, NSW, Australia; e Hunter Medical Research Institute, New Lambton Heights, NSW, Australia; g Mental Health Coordinating Council, Lilyfield, NSW, Australia; h Menzies Centre for Health Policy, School of Public Health, University of Sydney, Australia

**Keywords:** Community mental health services, Physical health, Health risk behavior, Chronic disease, Community organisation, Preventive care

## Abstract

People living with mental health conditions have a reduced life expectancy of approximately 10 years compared to the general population, largely due to physical chronic diseases and higher rates of tobacco smoking, poor nutrition, harmful alcohol consumption, physical inactivity and poor sleep behaviours. Community managed organisations (CMOs) may play a valuable role in providing preventive care to people with mental health conditions (consumers) to address these health behaviours. This paper reports the findings of a cross-sectional survey undertaken between November 2018 and February 2019 with leaders of CMOs (n = 76) that support people with mental health conditions in the state of New South Wales, Australia to: 1) measure the provision of preventive care (screening, support, and connections to specialist services) for five health behaviours; 2) identify the presence of key organisational features (e.g., data collection, staff training); and 3) explore if these organisational features were associated with the provision of preventive care. Preventive care provision to a majority of consumers (50% or more) was least frequently reported for tobacco smoking and most frequently reported for physical activity. Staff training and guidelines regarding the provision of preventive care were associated with the provision of such care. The results demonstrate that CMOs are already engaged in providing preventive care to some extent, with certain behaviours and preventive care elements addressed more frequently than others. Further research with additional CMO stakeholders, including staff and consumers, is needed to gain a deeper understanding of factors that may underlie CMOs capacity to routinely provide preventive care.

## Highlights

• Over 80% of CMOs are providing preventive care for at least one health behaviour.
• Between 16% and 57% of CMOs are providing preventive care for all health behaviours.
• Physical activity was most frequently addressed for 50% or more CMO consumers.
• Tobacco smoking was least frequently addressed for 50% or more CMO consumers.
• Staff training and guidelines were associated with preventive care provision.

## 1 Introduction

People living with mental health conditions experience a median of 10 years shorter life expectancy compared to the general population (Walker et al., 2015), largely due to a higher prevalence of chronic diseases such as cardiovascular diseases, chronic obstructive pulmonary disease and diabetes (Walker et al., 2015, Erlangsen et al., 2017). Such conditions are up to two times more prevalent among people with mental health conditions compared to the general population (Kilian et al., 2006, Harris., B, M, D, Batterham, P, Bartlem, K, Clinton-McHarg, T, Dunbar, J, Fehily, C, Lawrence, D, Morgan, M, and Rosenbaum, S, Australia's Mental and Physical Health Tracker: Background Paper, in issues paper no., 2018, Firth et al., 2019), a population group that has a higher prevalence of modifiable health behaviours including tobacco smoking, poor nutrition, harmful alcohol consumption, physical inactivity and poor sleep behaviour (Harris., B, M, D, Batterham, P, Bartlem, K, Clinton-McHarg, T, Dunbar, J, Fehily, C, Lawrence, D, Morgan, M, and Rosenbaum, S, Australia's Mental and Physical Health Tracker: Background Paper, in issues paper no., 2018, Firth et al., 2019, Stanley and Laugharne, 2014). Addressing multiple health behaviours among people with mental health conditions has been identified as critical to reducing the inequitable burden of chronic disease and extending life expectancy among this population (Firth et al., 2019, National Mental Health Commission, 2016, World Health Organisation, 2018, The Royal Australian and New Zealand College of Psychiatrists, 2015).

Brief interventions that aim to identify and address health behaviours (‘preventive care’) for people with mental health conditions are recommended in care delivery guidelines for public health and mental health settings internationally (The Royal Australian and New Zealand College of Psychiatrists, 2015, Canadian Mental Health Association, 2008, Public Health England, 2016) and within Australia (Department of Health & Human Services, 2019, National Mental Health Commission, 2016, Stanley and Laugharne, 2010, NSW Department of Health, 2017). Such guidelines typically recommend the provision of a number of elements of care: screening to identify health behaviours (e.g. whether a patient smokes); and support to modify health behaviours (e.g. providing brief advice and connections to specialist health behaviour change services) (Department of Health & Human Services, 2019, National Mental Health Commission, 2016, Stanley and Laugharne, 2010, The Royal Australian and New Zealand College of Psychiatrists, 2015, Public Health England, 2016, NSW Department of Health, 2017). The provision of all of these elements of preventive care (i.e. *comprehensive care*) compared to the provision of just one element, has been reported to be more effective for health behaviour change (Bartlem et al., 2019).

Although clinical practice guidelines do not specifically refer to community-managed organisations (CMOs); the potential of such organisations in delivering this care is well-recognised (Community Mental Health Australia, 2012, NSW Department of Health, 2017, Mental Health Coordinating Council and The Cancer Council, 2008, Hancock and Cowles, 2014). Within the context of the Australian mental health care system, CMOs are non-government organisations that deliver mental health programs and support services, characteristically partly-funded by government, to people living with mental health conditions (Australian Institute of Health and Welfare, 2019). CMOs provide a diverse range of psychosocial support and rehabilitation services including accommodation support, peer-support, employment and education, and physical health support (Community Mental Health Australia, 2012, Mental Health Coordinating Council, 2010) to address the holistic needs of consumers (Community Mental Health Australia, 2012); and have been suggested to be well-positioned to support people with their health behaviours through the provision of preventive care (Community Mental Health Australia, 2012, Mental Health Commission of NSW, 2020, NSW Department of Health, 2017, Mental Health Coordinating Council and The Cancer Council, 2008, Hancock and Cowles, 2014). While not reporting directly on the care provided, a systematic review of barriers and facilitators to the provision of evidence-based health and social care delivery interventions in non-government organisations identified a number of key factors that influence such intervention provision, including organisational culture, availability of resources, and monitoring and evaluation (Bach-Mortensen et al., 2018).

There is limited research that describes the type and prevalence of preventive care being routinely provided by CMOs; with research either focussing on the provision of care for one health behaviour (Bryant et al., 2012, Marlowe and Paynter, 2015, Mental Health Coordinating Council and The Cancer Council, 2008), or care addressing ‘physical health’ broadly rather than care with a preventive focus (Hancock and Cowles, 2014, Johnson and Fry, 2013). Therefore, a major gap in the published evidence is understanding the type and prevalence of preventive care being provided by CMOs to consumers, where this care addresses multiple health behaviours such as smoking, nutrition, alcohol consumption, physical activity, and sleep behaviours. Further, to date, no study has investigated the factors associated with the provision of preventive care for multiple health behaviours by CMOs that support people with mental health conditions.

Given the above evidence gaps, the aims of this study were to:

1. Describe the provision of preventive care to identify and address health behaviours among CMO consumers.

2. Identify the presence of certain organisational features (culture, resources, and monitoring and evaluation) known to facilitate implementation of evidence-based practices in CMOs, and explore their possible association with the provision of preventive care

## 2 Methods

### 2.1 Design and setting

A cross-sectional online survey was undertaken between November 2018 and February 2019 with leaders of CMOs that provide support to people with mental health conditions in New South Wales (NSW) Australia. NSW has the largest population in Australia (32% of Australian population; (Australian Bureau of Statistics, 2017) of whom approximately one-in-five live with a mental health condition (Australian Bureau of Statistics and Health, 2019). Ethics approval to conduct the research was obtained from the University of Newcastle Human Research Ethics Committee (H-2018-0354).

### 2.2 Participants

Eligible CMOs included any organisation which: was identified as a CMO or listed charity and provided care to adults (18 years of age or older) with mental health conditions (or their families or carers) in NSW. Organisational leaders of CMOs were eligible to participate if they: 1) were a CEO, Director or nominated senior executive level staff member for an eligible CMO; 2) were 18 years of age or older and; 3) had a valid email address.

### 2.3 Recruitment and data collection procedure

In the absence of a formalised list of CMOs in NSW, identification of potentially eligible organisations was facilitated using a directory of mental health services in the state [https://directory.wayahead.org.au/]). Web searches, phone calls and the member list of a NSW peak body for CMOs (Mental Health Coordinating Council [MHCC]) were used to clarify CMO status. Web searches and phone calls were conducted to obtain contact information for a senior leader of each identified potentially eligible organisation.

Identified leaders of potentially eligible CMOs received an email inviting them to participate in the study, including a study information sheet, a PDF copy of the survey, and a hyper-link to the online survey portal. To increase the response rate for the survey (Dillman et al., 2014) email reminders (at one and four weeks following the initial email invitation) and telephone reminders (at two weeks following the initial email invitation) were provided. The study was promoted through existing professional networks (MHCC and Mental Health Commission of NSW) via e-newsletters and webpages to increase awareness of and engagement in the study. All participants were entered into a draw to win a prize of $500 in value that could be used by their organisation towards purchasing resources or for staff to participate in training.

Participants could complete the survey via: an online survey portal, by hand and returning a scanned copy of the paper-version to the research team, or over the phone with a member of the research team. The survey was developed and distributed through the online platform ‘Qualtrics’ (Qualtrics, 2018) and took approximately 30 minutes to complete.

### 2.4 Measures

The survey items were developed for the purpose of this study due to the absence of validated measures available in the research area. Experts in the CMO sector, CMO leaders and the research team contributed to the development of the survey items through a process of consultation, revision and piloting to ensure content and face validity. Specific advice was sought from author CH (who had previously conducted self-report studies among CMO CEOs) regarding the level of detail that respondents could reliably report on. The survey was comprised of the following three sections:

#### 2.4.1 CMO service characteristics

Four items assessed the characteristics of the CMO: 1) service types delivered (e.g. staffed residential services); and number of 2) service locations, 3) staff members, and 4) consumers (see Table 1).

**Table 1:** Preventive care and organisational feature survey items (total of 55 items).

Survey items and response options	Health behaviours assessed
**Preventive care survey items (15 items)**	
***Health behaviour screening (5 items)*** Respondents were asked to estimate the proportion of consumers who were ‘routinely asked’ (defined as ‘asked systematically as part of standard practice or usual care’) about their^a^:	1) Tobacco smoking status 2) Nutrition 3) Physical activity 4) Alcohol consumption 5) Sleep behaviour
***Support to modify health behaviour (5 items)*** Respondents were asked to estimate the proportion of consumers that received support (defined as ‘either face-to-face, over the phone or online’; and possibly including ‘advice, information, programs or referrals’) from the CMO to^a^:	1) Reduce or quit tobacco smoking 2) Improve their nutrition 3) Reduce alcohol consumption 4) Improve physical activity 5) Improve sleep behaviours
***Connection to a specialist service (5 items)*** Respondents who reported that their organisation provided support to modify a particular health behaviour to 1% or more consumers were subsequently asked whether the support delivered included routinely providing connections to specialist services outside of the organisation for^b^: *Examples of external specialist services were provided for each health behaviour (*e.g. *an exercise physiologist in the case of improving physical activity)*	1) Reducing or quitting tobacco smoking 2) Improving nutrition 3) Reducing alcohol consumption 4) Improving physical activity 5) Improving sleep behaviours

**Organisational feature survey items (40 items)**	
***Organisational culture (10 items)*** Respondents were asked about: 1) Written policies: whether the organisation had a written policy regarding the provision of preventive care (e.g. formal policy, mission statement or key performance indicators) for^b^: 2) Guidelines: whether the organisation provided written guidelines to staff about the delivery of preventive care for^b^:	1) Reducing or quitting tobacco smoking 2) Improving nutrition 3) Reducing alcohol consumption 4) Improving physical activity 5) Improving sleep behaviours
***Resources (20 items)*** Respondents were asked about: 1) Funding: Whether the organisation had received funding for chronic disease prevention programs in the last 12 months that targeted^c^: 2) Dedicated staff role: Whether the organisation had employees whose roles were dedicated to addressing preventive care for^b^: 3) Staff training: The proportion of current staff that had received training to support consumers with^a^: 4) Tools or resources: Whether the organisation provided tools or resources to assist staff in supporting consumers with^b^:	1) Reducing or quitting tobacco smoking 2) Improving nutrition 3) Reducing alcohol consumption 4) Improving physical activity 5) Improving sleep behaviours
***Monitoring and evaluation (10 items)*** Respondents were asked about: 1) Data collection for prevalence of care provision: Whether the organisation collected data which allowed them to measure the proportion of consumers who received support for^b^: 2) Data collection for type of care provision: whether the organisation collected data which allowed them to measure the type of support provided to consumers for^b^:	1) Reducing or quitting tobacco smoking 2) Improving nutrition 3) Reducing alcohol consumption 4) Improving physical activity 5) Improving sleep behaviours

a Response options: 1. None (0%), 2. A few (1% − 24%), 3. Some (25% − 49%), 4. Most (50% − 74%), 5. Nearly all (75% − 99%), 6. All (100%), 7. Unsure.

b Response options: 1. Yes, 2. No, 3. Unsure.

c Response options: 1. No funding, 2. One-off funding, 3. Ongoing funding, 4. Both one-off and on-going funding, 5. Unsure.

#### 2.4.2 Preventive care provision

The provision of three preventive care elements were assessed separately for each health behaviour. These were: 1) the proportion of consumers provided with: health behaviour screening (‘None [0%]’ to ‘All [100%]’ consumers); 2) support to modify health behaviours (‘None [0%]’ to ‘All [100%]’ consumers); and 3) whether connections to specialist services were routinely provided as part of this support (‘Yes’, ‘No’ or ‘Unsure’). See Table 1 for the survey items.

#### 2.4.3 Organisational features

Three organisational features that have previously been identified as facilitators to the implementation of evidence-based practices in CMO settings (Bach-Mortensen et al., 2018) were assessed separately for each health behaviour: Organisational Culture – two questions (written policies, guidelines) for each of the five health behaviours ; Resources – four questions (funding, dedicated staff role, staff training, tools or resources) for each of the five health behaviours ; and Monitoring and Evaluation – two questions (data collected on care provision prevalence, data collected on care provision type) for each of the five health behaviours. See Table 1 for the survey items.

### 2.5 Statistical analysis

Data cleaning and analysis was completed using SPSS version 25.0 (IBM Corp, 2017). Descriptive statistics were used to describe the ‘CMO service characteristics’, ‘preventive care provision’ and ‘organisational features’ of responding CMOs. For descriptive analyses, ‘Organisational features’ were dichotomised (Yes/No) to indicate whether the feature was reported by the participant to be present or absent for each of the five health behaviours.

Given the importance of addressing all five health behaviours to reduce the burden of chronic disease for this population (Firth et al., 2019, O’Donoghue, 2021) each preventive care element was dichotomised as follows; 1) ‘health behaviour screening’ (provided to 1% or more consumers for all five health behaviours vs not provided to 1% or more consumers for all five behaviours); 2) ‘support to modify health behaviours’ (provided to 1% or more consumers for all five health behaviours vs not provided to 1% or more consumers for all five health behaviours); 3) ‘connection to a specialist service’ (provided for at least one health behaviour vs no connection provided for any health behaviours). These three dichotomised preventive care variables were also combined to form a ‘comprehensive care’ variable to identify CMOs providing all three preventive care elements.

The four preventive care variables (‘health behaviour screening’, ‘support to modify health behaviours’, ‘connection to a specialist service’, and ‘comprehensive care’) were entered as outcome variables into four separate logistic regression models to explore potential associations with organisational features. Organisational features were dichotomised to identify if each feature was present in the CMO for at least one health behaviour (vs absent for all behaviours). These seven ‘organisational features’ variables were entered into each logistic regression model as predictor variables for each of the four preventive care outcome variables created. For each model, a backward stepwise process was conducted until all remaining variables were significant (p-value less than 0.05). Odds ratios and 95% confidence intervals are reported.

## 3 Results

### 3.1 Sample characteristics

**Fig. 1:**
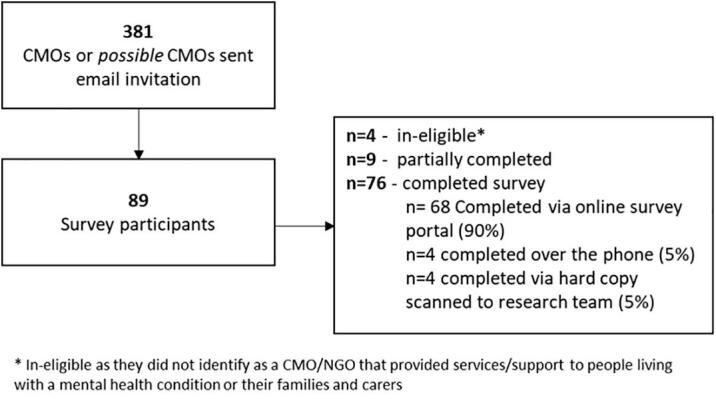
Flow diagram of surveyed participants.

One CEO or executive leader from each of the 381 CMOs identified as potentially eligible were invited to participate. The number of participating CMOs is presented in Fig. 1.

Table 2 reports characteristics of CMOs in NSW (n = 76) as reported by the participating organisational leader. Approximately 43% of CMOs had one service location, 31% provided support to between 1001 and 5000 consumers and 61% had less than 50 staff members. Most provided information and referral services (66%), group support activities (65%) and mental health awareness and promotion services (56%).

**Table 2:** Characteristics of CMOs in NSW (N = 76) as reported by the participating organisational leader.

Variables^a^	%	*n*
**Number of CMO locations**	*N* = 72	
Online presence only	4	3
1 location	43	31
2–10 locations	30	21
10 + locations	23	17
		
**Number of consumers with mental health conditions**	*N* = 74	
Less than 100	15	11
100–500	24	18
501–1000	12	9
1001–5000	31	24
5000+	18	12
		
**Number of staff members**	*N* = 76	
Less than 50	61	47
51–100	9	7
101–300	16	12
301–700	7	4
701–1000	5	4
1000–1500+	3	2
		
**Service types delivered**^b^	*N* = 76	
Counselling or helpline services	43	30
Information or referral services	66	50
Self-help services	22	17
Peer support or outreach services	45	32
Group support activities	65	49
Staffed residential services	22	17
Accommodation or housing support	34	28
Leisure or recreation support	28	22
Advocacy or representation services	45	35
Family support or carer services	40	31
Education, employment or training services	38	30
Physical health support	36	27
Mental health awareness and promotion	56	43
Mental illness prevention services	34	24

a N’s vary due to missing responses.

b Percentage does ≠ 100 as participants had the option to select more than one service type.

### 3.2 Preventive care provision

**Fig. 2:**
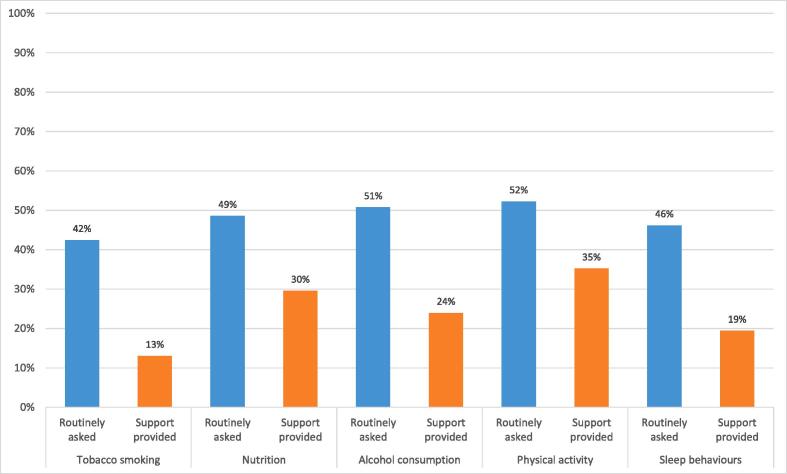
Proportion of responding CMOs providing 50% or more of consumers with preventive care (N = 76).

Two-fifths to half (42%−52%) of CMOs reported providing health behaviour screening (*routinely asking)* to 50% or more consumers for each health behaviour (Table 3*,* Fig. 2). Most CMOs (n = 64; 84%) reported providing health screening for at least one behaviour; and a majority provided health screening for all five health behaviours (n = 43; 57%) for at least some consumers (1% or more of consumers).

**Table 3:** Reported proportion of consumers estimated to be receiving preventive care from responding CMOs (N = 76).

	**Proportion of consumers estimated to be receiving preventive care**				
Variables	None (0%) consumers	A few (1%-24%) consumers	Some (25%-49%) consumers	Most (50%-74%) consumers	Nearly all/all (75%-100%) consumers
	% (*n*)	% (*n*)	% (*n*)	% (*n*)	% (*n*)
**Routinely asked about**^**a**^**:**					
Tobacco smoking status (*N* = 66)	25.8 (17)	22.7 (15)	9.1 (6)	15.2 (10)	27.3 (18)
Nutrition (*N* = 70)	15.7 (11)	24.3 (17)	11.4 (8)	20.0 (14)	28.6 (20)
Alcohol consumption (*N* = 67)	17.9 (12)	16.4 (11)	14.9 (10)	14.9 (10)	35.8 (24)
Physical activity (*N* = 67)	14.9 (10)	20.9 (14)	11.9 (8)	16.4 (11)	35.8 (24)
Sleep behaviour (*N* = 65)	18.5 (12)	18.5 (12)	16.9 (11)	23.1 (15)	23.1 (15)
At least one behaviour (N = 76)^b^	*–*	*84.2 (64)^c^*	–	–	–
All behaviours (N = 76)^bd^	*–*	*56.6 (43)^c^*	–	–	–

**Received support to**^**a**^**:**					
Reduce or quit tobacco smoking (N = 69)	36.2 (25)	34.8 (24)	15.9 (11)	5.8 (4)	7.2 (5)
Improve nutrition (N = 71)	21.1 (15)	22.5 (16)	26.8 (19)	14.1 (10)	15.5 (11)
Reduce alcohol consumption (N = 71)	29.6 (21)	32.4 (23)	14.1 (10)	14.1 (10)	9.9 (7)
Improve physical activity (N = 71)	19.7 (14)	25.4 (18)	19.7 (14)	9.9 (7)	25.4 (18)
Improve sleep behaviours (N = 67)	25.4 (17)	37.3 (25)	17.9 (12)	6.0 (4)	13.4 (9)
At least one behaviour (N = 76)^b^	–	*82.9 (63)^c^*	–	–	–
All behaviours (N = 76)^bd^	–	*44.7 (34)^c^*	–	–	–

^a^N’s vary due to missing responses and ‘unsure’ responses excluded.

^b^‘Unsure’ responses were recoded as ‘none 0%’ when calculating this variable.

^c^ Provision of care to at least some consumers (1% or more consumers).

^d^Outcome variable used in regression analysis.

Between 13% and 35% of CMOs reported providing support to modify health behaviours to a majority of consumers. Most CMOs (n = 63; 83%) reported providing support for at least one health behaviour, and less than half of CMOs reported providing support for all five health behaviours (n = 34; 45%) for at least some consumers (1% or more of consumers).

Connections to specialist services outside the organisation to support consumers with modifying health behaviours were least frequently provided for sleep (33% of CMOs), and most frequently provided for alcohol consumption (69% of CMOs), with approximately 50% of CMOs providing such connections for the other behaviours (nutrition 47%, tobacco smoking 48%, physical activity 53%). Most CMOs (82%) reported providing connections for at least one health behaviour: and 16% of CMOs reported providing connections for all five health behaviours.

Approximately 38% of CMOs (n = 29) reported providing at least some consumers with ‘health behaviour screening’ and ‘support to modify health behaviour’ for all five behaviours and at least one ‘connection to a specialist service’ (*comprehensive care*).

### 3.3 Organisational features

Most CMOs reported providing tools and resources to assist staff in providing preventive care (71%) and having staff members that had received training to provide preventive care (70%) (Table 4). Approximately half of CMOs reported having written policies (46%) or guidelines (45%) regarding the provision of preventive care. Slightly fewer CMOs reported collecting data regarding the provision of preventive care (41%) and having staff whose role was dedicated to addressing preventive care (38%). One in four CMOs (25%) reported receiving funding within the last 12 months for chronic disease prevention programs.

**Table 4:** Proportion of CMOs reporting the presence of organisational features for each health behaviour (N = 76).

Organisational Features^a^	Tobacco smoking N = 75		Nutrition N = 75		Alcohol consumption N = 76		Physical activity N = 76		Sleep behaviours N = 75		At least one behaviour^f^ N = 76		All behaviours N = 76	
	%	n	%	n	%	n	%	n	%	n	%	n	%	n
**Organisational Culture**														
Written policies:	31	23	34	25^b^	29	22	32	24^c^	18	13^b^	46	35	12	9
Guidelines:	28	21	33	24^c^	25	19	30	22^c^	17	12^d^	45	34	12	9

**Resources**														
Funding:	11	8^a^	11	8	11	8^c^	13	10	3	2^c^	25	19	1	1
Dedicated staff role:	24	18	24	18^b^	25	19^b^	25	19^b^	18	13^b^	38	29	15	11
Staff training:	52	39	53	40	53	40	55	42	45	34	70	53	40	30
Tools or resources:	51	38	61	46	48	36^b^	55	42	41	30^b^	71	54	30	23

**Monitoring and evaluation**														
Data collection^e^:	32	24	28	21	29	22	29	22	20	15	41	31	18	14

a N’s vary due to missing response.

b One missing response.

c Two missing responses.

d Three missing responses.

e Data collection for prevalence and/or type of preventive care provision.

f Predictor variables (n = 7) used in regression analysis.

### 3.4 Associations between organisational features and preventive care provision

The presence of CMO staff who received training to support consumers with at least one health behaviour increased the odds of: routinely asking (at least some consumers) about all health behaviours (OR = 3.7, 95% CI 1.3, 11.2, p = 0.014); and providing support (to at least some consumers) to modify all health behaviours (OR = 3.3, 95% CI 1.0, 11.1, p = 0.007), compared to CMOs without trained staff (Table 5). The odds of providing support to modify all health behaviours for at least some consumers were also increased by the presence of guidelines provided to CMO staff around the provision of preventive care (for at least one behaviour (OR = 5.0, 95% CI 1.8, 13.9, p = 0.002), compared to CMOs that did not provide such guidelines. The presence of guidelines also increased the odds of providing all elements of preventive care (i.e. comprehensive care; OR = 5.2, 95% CI 1.9, 14.3, p = 0.001) compared to CMOs without guidelines. There were no significant associations between any organisational features and the provision of a connection to a specialist service for at least one health behaviour.

**Table 5:** Associations between organisational features and the provision of preventive care (N = 76).

Outcome	Predictor	B	SE	OR	95% CI	df	*p*-value
Model 1. Routinely asked at least some consumers about all health behaviours							
	CMO staff received training^a^						
	Yes	1.29	0.53	3.65	(1.30,11.21)	1	0.014
	No (reference)	–	–	–	–	–	–

Model 2. Provided support to at least some consumers to modify all health behaviours							
	Guideline for preventive care^a^						
	Yes	1.61	0.52	5.01	(1.80,13.94)	1	0.002
	No (reference)	–	–	–	–	–	–
	CMO staff received training^a^						
	Yes	1.20	0.61	3.33	(1.00,11.10)	1	0.007
	No (reference)	–	–	–	–	–	–

Model 3. Comprehensive care for health behaviours							
	Guideline for preventive care^a^						
	Yes	1.66	0.51	5.24	(1.92,14.31)	1	0.001
	No (reference)	–	–	–	–	–	–

^a^Organisational feature present in CMO for at least one health behavior.

Note: There were no significant associations between organisational features and the provision of connections to specialist services.

## 4 Discussion

This is the first study to report the prevalence and characteristics of preventive care for multiple health behaviours among this population and setting. The results indicate that CMOs are providing variable levels of care for each preventive care element and health behaviour, and, that organisational features such as staff training and guidelines may be important to the implementation of preventive care in this setting. Whilst most CMOs were addressing at least one health behaviour, substantially less were addressing all five behaviours.

### 4.1 Preventive care provision

Across behaviours, approximately half of CMOs reported routinely asking a majority of consumers about their health behaviours, with the provision of this care element varying little between behaviours. This contrasts with a recent systematic review on preventive care provision across different mental health settings (i.e. primarily acute community and inpatient services) which identified substantial variation in the provision of asking about or assessing health behaviours; ranging from 62% of clients for alcohol consumption to 17% of clients for nutrition (Bailey et al., 2019). This contrasting result may be indicative of the psychosocial services delivered by CMOs where health promotion is a key element compared to public services which may be more focused on providing acute treatment to reduce distress (Community Mental Health Australia, 2012, Mental Health Coordinating Council, 2010, Australian Institute of Health and Welfare, 2021).

The proportion of CMOs providing support to modify health behaviours varied across health behaviours. Approximately one-third of CMOs reported providing a majority of consumers with support to modify physical activity and nutrition; whereas just over one-in-ten reported providing this support for tobacco smoking. The finding that screening occurred more frequently than health behaviour support is consistent with national (Bartlem et al., 2014, Bryant et al., 2012, Mental Health Coordinating Council and The Cancer Council, 2008) and international (Bailey et al., 2019, Shim et al., 2015, Brown, 2019, Otachi and Okoli, 2021) literature in community settings and may reflect a need for further action once a health concern is identified (Yarborough et al., 2018). Previous research among CMOs similarly identified that physical health support was largely focussed on improving nutrition and physical activity; typically consisting of cooking and food preparation sessions, grocery tours, walking groups and exercise sessions (Hancock and Cowles, 2014, Johnson and Fry, 2013). Qualitative evaluations of programs targeting physical activity and nutrition within CMOs identified important components to include: building health literacy (Ennals et al., 2020, Lo, 2014) and social connections (Ennals et al., 2020, Gill, 2012), peer-delivered support (Hancock and Cowles, 2014, Ennals et al., 2020, Lo, 2014, Gill, 2012) and partnerships between CMOs and other health services and organisations (Hancock and Cowles, 2014, Chapman et al., 2020).

Between one and two-thirds of CMOs reported routinely providing connections to specialist services to help consumers modify their health behaviours. Previous research across a range of community mental health settings has also focussed on connections with health services (including telephone support services, e.g. Quitlines) to improve care coordination and health promotion for consumers (Bryant et al., 2012, Chapman et al., 2020, Gerrity, 2014, Fortuna et al., 2020, Murphy et al., 2020, Scharf et al., 2016, McGinty et al., 2018, Ennals, 2019, Fehily et al., 2020, Bartlem et al., 2016, Daumit et al., 2020, Johnson et al., 2020). Evaluations of such programs have found improvements including: rates of screening (Fortuna et al., 2020, Murphy et al., 2020, Fehily et al., 2020), health service use (Fortuna et al., 2020), and cholesterol (Scharf et al., 2016) among participants. A qualitative study among leaders (N = 627) within US psychiatric rehabilitation settings (comparable to CMOs); identified key factors to the implementation of such programs including: fit with organisational culture, geographic proximity of health services, facilitating coordination with external providers and accessible Health IT (McGinty et al., 2018).

### 4.2 Organisational features, and associations with preventive care provision

Most CMOs reported having staff who were trained and had access to tools and resources to provide preventive care; however only one in four reported receiving funding within the last 12 months for programs that targeted at least one health behaviour. Given CMOs focus on addressing the holistic needs of consumers (Community Mental Health Australia, 2012), the provision of preventive care may be integrated in the process of supporting consumers in achieving their goals. However, with less than 40% of CMOs routinely asking the majority of consumers about health behaviours, a large proportion of consumers may be missing the opportunity to identify and receive support to address these behaviours. Future research should explore mechanisms to increase the routine and systematic provision of preventive care, including funding models that may assist CMOs to provide this care.

Approximately half of CMOs reported having policies or guidelines related to the provision of preventive care for at least one behaviour, and approximately 40% reported collecting data around the prevalence and/or type of preventive care provided for at least one health behaviour. Previous research in NSW CMOs has noted the importance of policies in promoting an environment that is conductive to discussing health behaviours (Mental Health Coordinating Council and The Cancer Council, 2008) and data collection in monitoring consumer progress with reaching health behaviour goals (Hancock and Cowles, 2014). Further, international literature in community mental health settings has similarly emphasised the importance of data collection systems to track and address unmet health needs of individuals (McGinty et al., 2018) and inform improvements of health programs (Annamalai et al., 2018).

The finding that staff training was associated with screening and support provision to at least some consumers to address all health behaviours was consistent with previous research in CMOs prioritising training as a key implementation strategy (Johnson and Fry, 2013, Marlowe and Paynter, 2015, Powell et al., 2015, Mental Health Coordinating Council and The Cancer Council, 2008, Hancock and Cowles, 2014) However, in order to improve the uptake of a practice, additional strategies should be considered such as assessing organisational readiness for change (pre-training) and providing ongoing technical support and consultation (post-training) (Stanhope et al., 2019). The presence of guidelines regarding preventive care provision was also significantly associated with the provision of support to modify all health behaviours and comprehensive care. Various factors influence the implementation of guidelines into routine practice, such as the complexity and level of awareness and support for the guideline by staff (Fischer et al., 2016). Although these organisational features appear to be important for the provision of preventive care within CMOs, further research to identify the types of training and guidelines that facilitate the systematic and routine provision of preventive care is needed.

### 4.3 Limitations

The primary limitation of the study is the low response rate (20%) limiting the generalisability of the study findings. Additional strategies could be utilised in future research such as increasing the number of contacts with participants (Dillman et al., 2014). Additional limitations of the study include 1) the small number of respondents indicating their CMO collected data regarding preventive care provision, potentially limiting the accuracy of estimates provided and 2) no information about the type of training staff received to address health behaviours. Further, due to the limited data available on NSW CMOs and the evolving environment in which CMOs operate, it is difficult to identify all existing CMOs at any point in time and therefore confidently identify a pool of eligible participants. Those organisations who did respond, however, appear to be similar to those who did not; a search of the websites of non-responding organisations found the most commonly reported service types provided were the same as those for responding organisations (i.e. information or referral services and group support activities). The current study also represents the largest sample of NSW CMOs to report on the prevalence of preventive care; with previous studies having only half the sample size of the current study (n = 35; (Hancock and Cowles, 2014), n = 38; (Mental Health Coordinating Council and The Cancer Council, 2008). An effort to establish a system that collects basic data on NSW CMOs active in this context is currently being undertaken by the MHCC and NSW Health; allowing more evaluation studies to be completed in the future (Mental Health Coordinating Council, 2019). Further research with a higher participation rate is required to confirm the findings and increase their generalisability.

### 4.4 Conclusions

CMOs are suggested to provide a conducive environment to address the health behaviours of people living with mental health conditions, with many organisations providing at least some consumers with an aspect of preventive care. Preventive care training and guidelines may be key strategies for CMOs to provide this care for all health behaviours. It is concerning that there appears to be less of a focus on smoking, as supporting cessation would likely have the biggest impact on the inequity in chronic disease. Further research including CMO consumers and staff is needed to gain a deeper understanding of the factors that underlie CMOs capacity to provide preventive care for different health behaviours.

## Funding

Funding for this research has been provided by The Australian Prevention Partnership Centre (G1800671) as part of the Boosting Preventive Health Research Program (Medical Research Future Fund [MRFF]).

## CRediT authorship contribution statement

**Lauren Gibson:** Conceptualization, Methodology, Formal analysis, Writing - original draft, Writing - review & editing. **Tara Clinton-McHarg:** Conceptualization, Methodology, Writing - review & editing, Supervision, Project administration, Funding acquisition. **Magdalena Wilczynska:** Conceptualization, Methodology, Writing - review & editing, Supervision. **Joanna Latter:** Conceptualization, Methodology, Writing - review & editing. **Kate Bartlem:** Writing - review & editing, Supervision, Funding acquisition. **Corinne Henderson:** Methodology, Writing - review & editing. **John Wiggers:** Writing - review & editing, Funding acquisition. **Andrew Wilson:** Writing - review & editing, Supervision, Funding acquisition. **Andrew Searles:** Writing - review & editing, Supervision, Funding acquisition. **Jenny Bowman:** Conceptualization, Methodology, Writing - review & editing, Supervision, Funding acquisition.

## Declaration of Competing Interest

The authors declare that they have no known competing financial interests or personal relationships that could have appeared to influence the work reported in this paper.
